# Brief Pain Inventory Pain Interference Subscale: Assessing Interference With Daily Living Activities in Older Adults With Multisite Musculoskeletal Pain

**DOI:** 10.3389/fpain.2022.897725

**Published:** 2022-05-09

**Authors:** Yael Koren, Suzanne G. Leveille, Tongjian You

**Affiliations:** ^1^Department of Nursing, Robert and Donna Manning College of Nursing and Health Sciences, University of Massachusetts, Boston, MA, United States; ^2^Department of Exercise and Health Sciences, Robert and Donna Manning College of Nursing and Health Sciences, University of Massachusetts, Boston, MA, United States

**Keywords:** pain interference, musculoskeletal pain, mobility, epidemiology, pain assessment, brief pain inventory, multisite pain

## Abstract

**Objectives:**

This study aims to determine domains of pain interference in daily routines assessed using the Brief Pain Inventory, in relation to multisite musculoskeletal pain among older adults living in the community.

**Design:**

The MOBILIZE Boston Study is a population-based study of 749 adults aged 70 and older.

**Measurements:**

Chronic musculoskeletal pain was assessed using a joint pain questionnaire and grouped as: no pain, single-site and multisite pain. The Brief Pain Inventory pain interference (PI) sub-scale assessed level of pain interference (0–10 rating) in 7 domains including general activity, mood, walking, work, relationships with people, sleep, and enjoyment of life. Interference ratings were grouped as: none (0), mild (>0 and ≤ 2), and moderate to severe (>2) PI.

**Results:**

PI was more common among women and those with less education compared to others. Older adults with chronic conditions such as osteoarthritis, depression, spinal stenosis, peripheral artery disease, and asthma/lung disease were more likely than their peers to report PI (*p* < 0.05). Multisite musculoskeletal pain was strongly associated with pain interference in all domains (*p* < 0.05). More than half of older adults with multisite musculoskeletal pain reported moderate to severe PI with general activity and walking. The highest prevalence of moderate to severe PI (score >2) in general activity was seen in participants with depression (62%), knee and hand osteoarthritis (71%) and peripheral artery disease (65%).

**Conclusion:**

Greater attention to PI and PI domains such as general activity and walking, could aid in efforts to reduce the overall impact of multisite musculoskeletal pain among older adults.

## Introduction

Almost half of older adults experience chronic multisite musculoskeletal pain (MMP) contributing to difficulty in their daily activities (1–3). Yet, little is known about specific domains by which MMP pain interferes in daily living. Pain interference (PI) describes the influence of pain on daily living and is distinct from pain severity and other characteristics of pain. Additionally, pain interference is a strong predictor of disability and falls, and a contributor to psychological concerns related to falls (4–6). Therefore, a better understanding of the characteristic of pain interference, or the ways by which pain interferes with older adults' daily activities is warranted. This study aims to determine domains of pain interference related to MMP in a cohort of older adults living in the community.

## Methods

### Participants

The Maintenance and Balance, Independent Living, Intellect and Zest in the Elderly (MOBILIZE) study is a population-based study of 765 older adults living in the community in the Boston area. Participants were recruited door to door in Boston and nearby suburbs based on city and town lists of people aged 70 and older living in the community (7). The demographic characteristics of the sample were comparable to the US 2000 Census for the Boston area (7). For this study, we used baseline data collected from 2005 to 2008 in a 2-part assessment involving an in-home interview followed by a clinic-based examination. Study eligibility criteria included age 70 years old or older, able to communicate in English and walk 20 feet without help from another person and plans to remain in the area for the subsequent 2 years. Participants were excluded for diagnosis of a terminal disease, moderate to severe cognitive impairment (Mini-mental State Examination <18) and severe vision and hearing deficits. Although a small number of spouses and partners who were 65 years old and older (*n* = 16) who were otherwise eligible were enrolled, the sample for this study was restricted to those aged 70 and older (*n* = 749). The institutional review boards of Hebrew SeniorLife and the University of Massachusetts Boston approved all protocols for the study. Detailed study methods were published previously (7).

### Measurements

The Brief Pain Inventory (BPI) subscale was used to assess the level of pain interference (PI) in the previous week in the following 7 domains: general activity, mood, walking, work, relations with others, sleep, and enjoyment of life (8). Participants rated the level of interference in each domain using a 0–10 numeric rating scale, with 0 indicating no interference and 10, completely interferes. The PI subscale score is the average of the 7 ratings. The BPI had been shown to be reliable and valid with non-cancer pain such as back pain, arthritis and peripheral neuropathy (9, 10). Musculoskeletal pain distribution was assessed using a joint pain questionnaire, assessing pain present for 3 or more months in the past year and present in the previous month in 6 areas: back, hands/wrists, shoulders, hips, knees, and feet (4). The musculoskeletal pain distribution was then classified as no pain, single site pain, and multisite pain (≥2 sites). This measure has been shown to predict disability and falls in the MOBILIZE Boston cohort (4, 11).

We considered pain interference in relation to several sociodemographic characteristics and chronic conditions. Age, gender, race, and education were assessed in the home interview. Additionally, chronic conditions such as spinal stenosis, asthma and lung disease were self-reported in the home interview. Heart disease was assessed based on self-reported history of a myocardial infarction or congestive heart failure, or presence of angina using the Rose Angina Questionnaire (12). Peripheral artery disease (PAD) was determined using the ankle-arm index (<0.90) and the Rose intermittent claudication questionnaire (12). Peripheral neuropathy was assessed with the Semmes-Weinstein monofilament testing (7, 13). Depression was measured based on the Hopkins revised Center for Epidemiologic Studies Depression Scale (CESD-r) (14). Diabetes was assessed using a disease algorithm based on self-reported diabetes, use of insulin or oral hypoglycemics and laboratory measures, random glucose (≥200) and hemoglobin A1C (>7%) (4). During the clinic examination, osteoarthritis was assessed by certified research nurses using the American College of Rheumatology (ACR) clinical criteria for knee and hand osteoarthritis (15, 16). Body mass index (BMI) was calculated using measured height and weight assessed in the clinic examination.

### Data Analysis

Presence of pain interference (score >0) vs. no pain interference (score = 0) was examined according to demographic characteristics and health conditions using descriptive statistics and chi square tests. Similarly, BPI pain interference was examined according to musculoskeletal pain distribution using chi square tests for between group differences and descriptive statistics, For the purpose of this analysis, the PI ratings were grouped according to: none (0), mild (>0 and ≤ 2), and moderate to severe (>2) (4). *P*-values <0.05 were considered significant. Additionally, we assessed correlations between the 7 BPI domains. Correlations >0.6 were considered strong correlations and correlations >0.4 and ≤ 0.6 were considered moderate correlations (17). Analysis was conducted using SAS v9.4 (SAS Institute Inc., Cary, NC, USA).

## Results

Overall, 462 participants (67%) reported pain interference. Age and race were not associated with pain interference (Table 1). PI was more common among women and those with less education (*p* = 0.03 and *p* = 0.01, respectively). A number of chronic conditions were associated with PI including hand and knee osteoarthritis, depression, spinal stenosis, and asthma/ lung disease (*p* < 0.005).

**Table 1:** Participant characteristics according to pain interference (PI), MOBILIZE Boston Study, 2005–2007.

	**No PI (*n =* 284)**	**PI (*n =* 462)**	***P*** ^*^
	***n*** **(%)**	***n*** **(%)**	
**Age**			
70–74 y	82 (28.9)	135 (29.2)	0.999
75–79 y	93 (32.8)	150 (32.5)	
80–85	68 (24.0)	112 (24.2)	
>85 y	41 (14.4)	65 (14.1)	
**Gender**			0.025
Female	165 (58.1)	306 (66.2)	
Male	119 (41.9)	156 (33.8)	
**Race**
White	227 (79.9)	350 (75.9)	0.43
Black	41 (14.4)	82 (17.8)	
Other	16 (5.6)	29 (6.3)	
**Body mass index**			0.68
<25	88 (31.5)	128 (28.5)	
25–29	117 (41.9)	198 (44.1)	
≥30	74 (26.5)	123 (27.4)	
**Education**			0.013
High school	20 (7.0)	65 (14.1)	
High school graduate	71 (25.0)	103 (22.3)	
College graduate	193 (68.0)	293 (63.6)	
**Asthma/Lung disease**	32 (11.3)	87 (19.0)	0.005
**Depression**			0.005
Minor depression	10 (3.5)	41 (8.7)	
Major depression	0 (0)	4 (0.9)	
**Heart disease**	113 (39.8)	200 (43.3)	0.29
**Diabetes**	50 (17.6)	100 (21.7)	0.18
**Spinal stenosis**	31 (10.9)	107 (23.2)	<0.001
**Osteoarthritis**			<0.001
Knee	26 (9.2)	106 (23.0)	
Hand	25 (8.8)	61 (13.2)	
Both	4 (1.4)	54 (11.7)	
**Neuropathy**	33 (11.70)	59 (13.0)	0.60
**Peripheral Artery disease (PAD)**	10 (3.5)	62 (13.4)	<0.001

*Pain interference measured using the Brief Pain Inventory; presence of PI determined as PI summary score >0*.

* *Chi-square test for between-group differences, significant p ≤ 0.05*.

**Figure 1:**
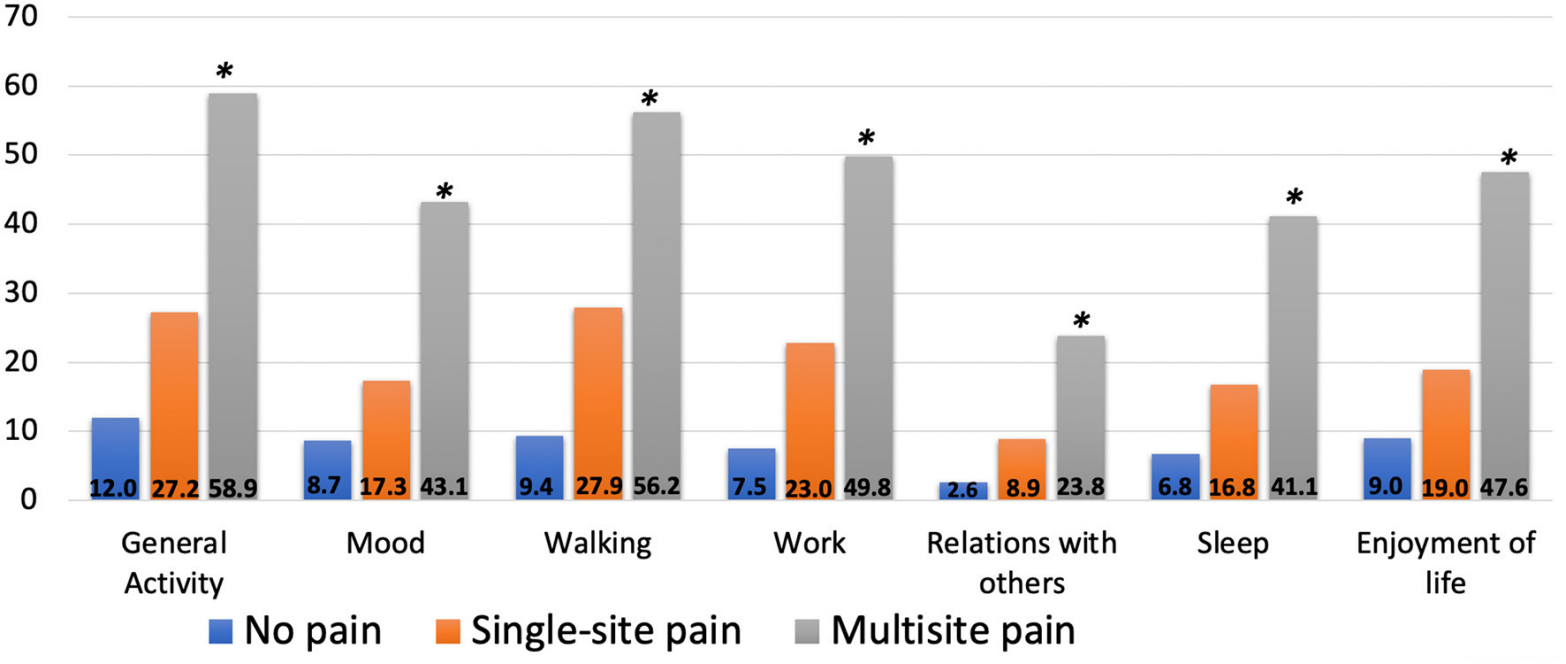
Prevalence of moderate-severe pain interference by domain according to pain distribution, MOBILIZE Boston study. *Chi-square test for between-group differences, *p* < 0.001.

Multisite pain and single site pain, reported by 40 and 24%, respectively, were strongly associated with all domains of PI. Overall, twice as many participants with multisite pain reported PI compared to those with single-site pain (Figure 1). The domains of PI most strongly associated with pain distribution were general activity and walking, with more than half of participants with MMP reporting moderate to severe interference in these areas. The domain, relations with others, had the lowest prevalence of reported moderate to severe interference, though the association with pain distribution was strong. For example, among those with multisite pain, 24% reported moderate to severe PI in their relations with others compared with 9% of those with single site pain.

A number of chronic conditions were associated with domains of PI (Table 2). The highest prevalence of moderate to severe PI (score >2) in general activity was seen in participants with depression (62%), knee and hand OA (71%) and PAD (65%). Similarly, participants with these conditions had highest rates of PI in walking and working as well. In general, participants with both hand and knee OA had the highest prevalence of moderate to severe PI across domains. Additionally, we found that depression was the only condition where more than half of participants reported moderate or severe PI in 6 of the 7 domains. The lowest prevalence of moderate to severe PI across all chronic conditions was seen in relations with others (15–36%) followed by PI in mood (23–53%).

**Table 2:** Prevalence of moderate-severe PI (>2 PI) according to chronic conditions, MOBILIZE Boston study.

	**Prevalence in sample** ***n*** **(%)**	**General activity** ***n*** **(%)**	**Mood** ***n*** **(%)**	**Walking** ***n*** **(%)**	**Working** ***n*** **(%)**	**Relationships** ***n*** **(%)**	**Sleep** ***n*** **(%)**	**Enjoyment of life** ***n*** **(%)**
Depression	55 (7)	34 (62)	29 (53)	31 (56)	30 (55)	13 (27)	27(55)	34 (62)
Diabetes	150 (20)	68 (45)	48 (32)	58 (39)	52 (35)	14 (19)	42 (28)	58 (39)
Heart disease	313 (42)	122 (39)	91 (29)	109 (35)	98 (32)	47 (15)	84 (27)	95 (30)
Spinal stenosis	138 (19)	69 (50)	47 (34)	64 (47)	68 (50)	25 (18)	50 (36)	59 (43)
Arthritis								
ss Knee	132 (18)	60 (46)	41 (31)	70(53)	49 (37)	20 (15)	38 (29)	41 (31)
Hand	86 (12)	37 (43)	20 (23)	34 (40)	32 (37)	15 (18)	25 (29)	31 (36)
Both	58 (8)	41 (71)	31 (53)	39 (67)	35 (62)	21 (36)	32 (55)	35 (60)
Lung disease	119 (16)	58 (49)	44 (37)	58 (49)	51(43)	26 (22)	43 (36)	44 (37)
Neuropathy	92 (13)	38 (41)	26 (28)	38 (41)	32 (35)	16 (17)	25 (28)	35 (38)
Peripheral artery disease	72 (10)	47 (65)	27 (38)	45 (63)	43 (60)	18 (25)	28 (39)	34 (47)

We evaluated correlations between the 7 domains and found that all were moderately to strongly correlated (data not shown). Pain interference in general activity was strongly correlated with PI in mood and working (*r* = 0.74 and *r* = 0.76 respectively). Also, PI in walking was strongly correlated with PI in working (*r* = 0.75). Sleep was moderately correlated with each of the other PI domains (*r* < 0.6). Among all correlations between sleep and the 6 domains, PI in walking and sleep had the lowest correlation (*r* = 0.439).

In an additional exploratory analysis based on demographic differences shown in Table 1, we observed a number of differences in prevalence of PI within domains according to gender and education (Table 3). Higher proportions of women than men consistently reported moderate to severe pain interference in the 7 domains. For example, ~45% of women reported moderate to severe pain interference in general activity, compared to ~26% of men (*p* < 0.05). Among both genders, pain interference in general activity and walking had highest prevalence; with ≥37% of women and ≥26% of men reporting PI in these domains. According to education levels, we found that, compared to those with more education, participants with less education had a higher prevalence of pain interference in all 7 pain interference domains. For example, ~57% of participants with less than high school education reported pain interference in the general activity domain compared to 36% of those with high school education and 30% of those with college education (*p* < 0.05).

**Table 3:** Prevalence of moderate to severe pain interference by domains according to gender and education in older adults^*^.

	**General activity**	**Mood**	**Walking**	**Working**	**Relationships**	**Sleep**	**Enjoyment of life**
	**Percent**						
Gender							
Female	44.7	27.9	36.8	32.8	14.9	26.2	29.8
Male	25.2	18.9	25.8	20.2	9.1	17.4	22.2
Education level							
< High school	56.5	37.7	55.3	45.2	24.7	34.1	42.9
High school graduate	36.2	29.3	33.3	34.5	16.1	27.2	29.3
College Graduate	30.1	20.5	28.7	22.9	9.5	19.6	23.3

^*^Chi square test for between group differences, Gender (p < 0.05), Education (p ≤ 0.01).

## Discussion

Pain interference with daily life is a pervasive problem, affecting two-thirds of the older population, and is especially burdensome among those who have MMP. Our results suggest that pain interference as a summary score does not reveal the full picture of the ways through which pain interferes with daily living. Domains such as walking and general activity are more substantially impacted than other domains such as relationships. Our findings speak to the variability in the daily burden of chronic pain and pain-associated chronic conditions among older adults. Reports of PI differed among older adults based on gender, education, and presence of a number of chronic conditions (arthritis, depression, spinal stenosis, lung disease, and peripheral artery disease).

Pain interference is one characteristic of pain that often is not included in routine pain assessments in older adults. Instead, pain assessments often rely on ratings of pain severity and identifying singular pain locations (25, 27, 28). Multisite pain stands out as a global measure of pain predictive of disability and falls (4, 5). Our results support the call for greater attention to pain distribution and specifically, the problem of MMP pain in older adults (2). The traditional focus on identifying and treating specific sites of pain in the clinical setting has neglected the overall functional burden of MMP. Our results may lead to a further study to better understand the observed associations of MMP across domains of pain interference but with greatest detriment in the areas of walking and general activity. Problems with walking and mobility in general contribute to loss of independence in the older population, thus it is critically important that pain management approaches for older adults with MMP effectively target these key functional areas.

Our findings showing the prevalence of moderate to severe PI in chronic conditions are consistent with previous reports on the disabling impact of chronic conditions. For example, the high prevalence of pain interference among those with PAD specifically in the general activity, walking, and working domains, is consistent with previous studies showing severity of walking-related disability in people with PAD (20, 24). With respect to the high prevalence of PI among people with depression, several studies have reported on the inter-relatedness and bidirectionality of pain and depression (19, 26, 29). Arola et al. prospectively studied the relation between depression and pain among community dwelling participants aged 50 or older and reported on their reciprocal relationship, supporting the need for clinically screening for PI (26). This is particularly important in older adults because of the high co-occurrence of these conditions and the likelihood for pain interference and its association with functional limitations in this population (29).

Our findings regarding the highest prevalence of PI reported in walking, general activity and work are expected because of the mobility limitations and disability associated with chronic pain (5, 30). Very few studies have evaluated prevalence of the 7 domains of PI among community dwelling older adults. It is interesting to note the low prevalence of moderate to severe pain interference according to pain distribution in the social domain of relations with others. Similar findings have been confirmed by Zhou et al. (18) who found that older adults residing in southern China reported that pain mildly interferes with their relations with others compared to moderate interference noted in general activity, walking and mood (18). One possible explanation could be that in old age, there may be fewer social expectations and obligations, and therefore less focus on the way that pain is impacting social relationships (21). Worse pain severity may also play a role in reported pain interference with relationships (21). For example, a longitudinal study by Yang et al. examined the relationship between pain severity and friendship and reported that new onset of severe pain later in life predicted fewer friendships than those with a new onset of moderate pain (21). In our current study, moderate to severe pain were grouped together and further understanding differences in PI across pain severity ratings is needed. In contrast, previous studies among adults (median age 62) and older adults have confirmed the negative impact that pain can have on an individual's relations with other people, friendship quality and partner interactions (22, 23). Despite the association between pain and psychological distress, our results suggest that although pain may interfere with relations, it may be less burdensome on social relationships than physical activities such as walking and general activity which are central to maintaining independence. Further research is needed to better understand the relationship between musculoskeletal pain and social engagement in older adults.

Our study design brings a number of strengths to our study. The representative sample of the MOBILIZE Boston study cohort of older community-dwelling adults strengthens the generalizability of the current findings to the English-speaking population of older adults living in the urban-suburban communities of the Northeast U.S. Additionally, the use of validated scales such as the Brief Pain Inventory Scale (BPI) and Center for Epidemiologic Studies Depression Scale (CESD-r) as well as clinical assessments and objective measures are important strengths of the study. Implications of our findings are limited by the cross-sectional design which cannot determine directionality or temporality of the observed relationships. The data were collected in 2005–2008 but the findings from this cohort continue to be very relevant given the continuing burden of multisite pain in the increasingly older population of the US (1). Future studies are needed using multivariable methods to determine the possible impact of confounding on these results. Longitudinal approaches could determine patterns of pain interference over time and interventional studies are needed to modify the impact of pain and interference on daily life in older persons.

## Conclusion

In sum, pain interference is an important indicator of the impact of pain on daily living in older adults, especially those with MMP. Our findings suggests that pain management programs for older adults with MMP could incorporate measures of pain interference in monitoring the effectiveness of their interventions particularly with attention to specific domains of pain interference. This approach could enhance our understanding of the benefits of specific pharmacologic and non-pharmacologic approaches to pain management. For example, the benefits of exercise for pain management may be more evident if pain interference was included as a target of treatment. Further research is needed to understand the factors contributing to pain interference as a distinct characteristic of pain in older adults in order to effectively intervene and treat MMP.

## Data Availability Statement

The datasets presented in this study can be found in online repositories. The names of the repository/repositories and accession number(s) can be found below: https://ifar-dataverse.hsl.harvard.edu/dataverse/mobilize.

## Ethics Statement

The studies involving human participants were reviewed and approved by University of Massachusetts Boston IRB and Hebrew SeniorLife IRB. The participants provided their written informed consent to participate in this study.

## Author Contributions

YK and SL conducted the data analysis. All authors contributed substantially to the manuscript writing. All authors contributed to the article and approved the submitted version.

## Funding

This work was supported by National Institute on Aging grants: #R56AG062737, #R01AG041525, and #P01AG004390.

## Conflict of Interest

The authors declare that the research was conducted in the absence of any commercial or financial relationships that could be construed as a potential conflict of interest.

## Publisher's Note

All claims expressed in this article are solely those of the authors and do not necessarily represent those of their affiliated organizations, or those of the publisher, the editors and the reviewers. Any product that may be evaluated in this article, or claim that may be made by its manufacturer, is not guaranteed or endorsed by the publisher.
